# Human Cytomegalovirus MicroRNAs miR-US5-1 and miR-UL112-3p Block Proinflammatory Cytokine Production in Response to NF-κB-Activating Factors through Direct Downregulation of IKKα and IKKβ

**DOI:** 10.1128/mBio.00109-17

**Published:** 2017-03-07

**Authors:** Meaghan H. Hancock, Lauren M. Hook, Jennifer Mitchell, Jay A. Nelson

**Affiliations:** Vaccine and Gene Therapy Institute, Oregon Health and Science University, Beaverton, Oregon, USA; Columbia University Medical College

## Abstract

Emerging evidence indicates that human cytomegalovirus (HCMV) manipulates host cell signaling pathways using both proteins and noncoding RNAs. Several studies have shown that HCMV induces NF-κB signaling early in infection, resulting in the induction of antiviral proinflammatory cytokines with a subsequent reduction of these cytokines late in infection. The mechanism for late cytokine reduction is unknown. In this study, we show that HCMV microRNAs (miRNAs) miR-US5-1 and miR-UL112-3p target the IκB kinase (IKK) complex components IKKα and IKKβ to limit production of proinflammatory cytokines in response to interleukin 1β (IL-1β) and tumor necrosis factor alpha (TNF-α). Transfection of miR-UL112-3p and miR-US5-1 mimics reduced endogenous IKKα and IKKβ protein levels, and site-directed mutagenesis of the 3′ untranslated regions (UTRs) identified the binding sites for each miRNA. Infection with mutant viruses lacking these miRNAs resulted in increased levels of IKKα and IKKβ proteins, an impaired ability to control NF-κB signaling at late times of lytic infection, and increased production of proinflammatory cytokines compared to wild-type virus in cell types relevant to HCMV infection *in vivo*. These phenotypes were rescued by preexpression of miR-US5-1 and miR-UL112-3p in infected cells or by a miR-US5-1/miR-UL112-3p double mutant virus that expresses short hairpin RNAs (shRNAs) targeting IKKα and IKKβ, demonstrating the gene specificity of the miRNAs. These observations describe a mechanism through which HCMV miRNAs expressed late in the infectious cycle downregulate proinflammatory cytokine production to create a cellular proviral environment.

## IMPORTANCE

Human cytomegalovirus (HCMV) is a significant cause of morbidity and mortality in transplant recipients and causes hearing loss and mental retardation when acquired congenitally. Initial events during HCMV infection result in the activation of NF-κB signaling, which culminates in the production of IL-6, CCL5, and TNF-α. Several viruses have developed mechanisms to block the antiviral effects of these cytokines. We show here that two HCMV miRNAs, miR-US5-1 and miR-UL112-3p, specifically downregulate IKKα and IKKβ signaling factors necessary to propagate NF-κB signaling and subsequent IL-6, CCL5, and TNF-α production. Regulation of these proinflammatory cytokines during lytic infection and during latency is critical to viral survival in the host.

## INTRODUCTION

The proinflammatory cytokines interleukin 1β (IL-1β) and tumor necrosis factor alpha (TNF-α) play essential roles at the interface of the innate and adaptive immune responses (1). IL-1β and TNF-α are involved in many key cellular processes, including proliferation, differentiation, apoptosis, and antiviral responses. These cytokines bind to specific cell surface receptors (IL-1R for IL-1β and TNFR1 or TNFR2 for TNF-α) after release from activated macrophages and/or T cells at the site of infection. Receptor-ligand interactions induce the formation of receptor-proximal multiprotein complexes that mediate signaling to downstream effector kinases and ubiquitin ligases. IL-1β and TNF-α signaling pathways converge at the activation of the IκB kinase (IKK) complex and subsequent release of the NF-κB transcription factors (2). Activation of NF-κB signaling induces the expression of proinflammatory cytokines and chemokines as well as adhesion and costimulatory molecules (3) and plays a key role in shaping the innate and adaptive immune responses.

NF-κB signaling is tightly regulated at the level of IκB phosphorylation. The IKK complex, composed of IKKα, IKKβ, and IKKγ (NEMO), is activated by phosphorylation of IKKα or IKKβ on serine residues within their activation loops either by upstream kinases or through autophosphorylation (4). The activated complex goes on to phosphorylate IκBα, causing its ubiquitin-mediated degradation (5, 6) and release of the NF-κB subunits. In the canonical NF-κB pathway, linear ubiquitination of NEMO assembles the IKK complex (7) and ultimately results in release of the p50 and RelA (p65) subunits that subsequently translocate to the nucleus, where they can bind to κB binding sites in the promoters of regulated genes (5). Noncanonical NF-κB signaling is mediated by activation of IKKα and results in release of p52 and RelB heterodimers. The timely activation of the IKK complex downstream of TNF-α and IL-1β receptor binding is the single most important regulatory step in the induction of NF-κB-mediated transcription.

The importance of the NF-κB signaling axis to limiting viral replication and spread is highlighted by the many pathogens that act to modulate this pathway. Human cytomegalovirus (HCMV) is a ubiquitous betaherpesvirus that has coevolved extensively with its host, resulting in a complex relationship between HCMV and the NF-κB signaling pathway. Activation of NF-κB signaling during HCMV infection is initiated by viral binding and entry (8–10), and many groups have demonstrated that NF-κB signaling is required for efficient transactivation of the major immediate early promoter (MIEP) of HCMV (11–15), especially in quiescent cells (16). The virus also dampens the antiviral responses elicited by exogenous stimulation of the NF-κB signaling pathways at later times in infection (17–19) and encodes several proteins that work to block different aspects of the NF-κB signaling pathways (20, 21). For example, IE86 blocks TNF-α-induced NF-κB signaling and activation of target gene transcription (21), while the tegument protein UL26 blocks TNF-α-induced IKK phosphorylation through unknown mechanisms (20). HCMV also partially blocks TNF-α signaling through downregulation of TNFR1 surface expression during lytic infection (22), while IL-1β- and TNF-α-induced expression of the chemokine monocyte chemoattractant protein 1 (MCP-1) is blocked at the level of transcription (23). Highlighting the complex relationship between HCMV and NF-κB signaling, the virus also encodes proteins that can activate or enhance NF-κB signaling. The UL144 protein activates NF-κB signaling through interactions with TNFR-associated factor 6 (TRAF6) and TRIM23 (24–26). UL138, one of a limited number of proteins expressed during HCMV latency in CD34^+^ hematopoietic progenitor cells (HPCs) (27), binds TNFR1 and enhances surface density and signaling from the receptor (28, 29). The complexity surrounding the regulation of NF-κB signaling during HCMV infection likely relates to the opposing interests of the virus depending on the cell type infected and the stage of the viral life cycle, especially *in vivo*. It is possible that the activation of NF-κB signaling and production of proinflammatory cytokines and chemokines are important for viral dissemination by recruiting cells to the site of infection. Likewise, enhancing cell surface expression of TNFR1 during latency may poise the virus for reactivation of the MIEP. However, given that the virus encodes multiple proteins that act to limit NF-κB signaling, there are times during its life cycle, likely during the establishment of latency, when the virus must decrease the production of proinflammatory and antiviral factors.

One means by which NF-κB signaling can be blocked is through the actions of cellular and viral microRNAs (miRNAs). miRNAs are short, 20- to 24-nucleotide noncoding RNAs that posttranscriptionally regulate gene expression by targeting the RNA-induced silencing complex (RISC) to mRNAs containing partially complementary sequences. Depending on the amount of complementarity between the target mRNA and the RISC-loaded miRNA, transcripts either are immediately degraded or undergo translational inhibition, often followed by mRNA degradation (30). miRNAs require only 6 to 8 nucleotides of complementarity within their “seed” region, and as such, one miRNA can potentially target up to 200 different transcripts (31). HCMV carries at least 22 mature miRNAs (32) whose expression increases during lytic infection in fibroblasts (33). Given their nonimmunogenic nature, miRNAs may provide a relatively robust means to silence host and viral gene expression during both lytic and latent infections.

Several studies have shown that gammaherpesvirus miRNAs target components of the NF-κB signaling pathway to enhance viral replication or to maintain latency. Kaposi’s sarcoma-associated herpesvirus (KSHV) miR-K5 and miR-K9 target MyD88 and IRAK1, respectively, to limit release of proinflammatory cytokines (34). Conversely, KSHV miR-K1 targets the inhibitor IκBα, which is important to prevent lytic replication in 293T and PEL cells (35). Epstein-Barr virus (EBV) miR-BART3 and miR-BART1 disrupt NF-κB signaling, potentially through targeting CAND1 and FBXW9 (36).

Our group and others have shown that HCMV miRNAs target both viral and cellular transcripts to aid in productive infection and persistence. HCMV miR-UL112-3p targets the 3′ untranslated region (UTR) of the major transactivator immediate early 72 (IE72) gene (37). Deletion of the miR-UL112-3p target site in the IE72 transcript causes an increase in IE72 expression (38), suggesting a possible role for miR-UL112-3p in latency establishment. In addition, targeting of IE72 may help prevent T cell recognition of latently infected cells (39). HCMV miRNAs also act to manipulate innate immune signaling pathways. Major histocompatibility complex (MHC) class I-related chain B (MICB) is a stress-induced ligand for the NKG2C on NK cells and is a common target of HCMV, EBV, and KSHV miRNAs (40, 41). HCMV miRNAs miR-US5-1, miR-US5-2, and miR-UL112-3p target multiple cellular genes within the endocytic recycling pathway to allow for efficient virion assembly compartment formation and to limit the release of proinflammatory cytokines (42), while miR-UL148D-1 targets ACVR1B to limit IL-6 secretion (43) and targets RANTES/CCL5 directly (44). Additionally, we have previously shown that miR-UL112-3p targets the Toll-like receptor 2 (TLR2) transcript to limit signaling through the TLR2-IRAK1 axis (45), suggesting that NF-κB signaling may also be a target of HCMV miRNAs.

Here, we provide evidence that the HCMV miRNAs miR-US5-1 and miR-UL112-3p play critical roles in dampening NF-κB signaling during the later stages of lytic infection in order to limit the release of proinflammatory cytokines. We demonstrate that miR-US5-1 and miR-UL112-3p directly target the IKK complex kinases IKKα and IKKβ and identify the miRNA binding sites within their 3′ untranslated regions (UTRs). Viral mutants lacking these miRNAs do not control NF-κB signaling as efficiently as wild-type (WT) virus, which results in increased proinflammatory cytokine production. Expression of short hairpin RNAs (shRNAs) targeting IKKα and IKKβ in the context of a viral mutant lacking miR-US5-1 and miR-UL112-3p is able to restore proinflammatory cytokine production to levels observed during WT virus infection, indicating that the observed phenotype is due to targeting of IKKα and IKKβ and not other known or unknown miRNA-targeted proteins. Modulation of NF-κB signaling by viral miRNAs in concert with viral proteins underscores the myriad ways in which HCMV manipulates host signaling pathways to aid in its replication. By interfering with the production of proinflammatory cytokines through manipulation of the NF-κB signaling pathway, HCMV miRNAs play an important role in shaping the antiviral immune response *in vivo*.

## RESULTS

### HCMV miRNAs partially block signaling mediated by IL-1β and TNF-α.

**FIG 1:**
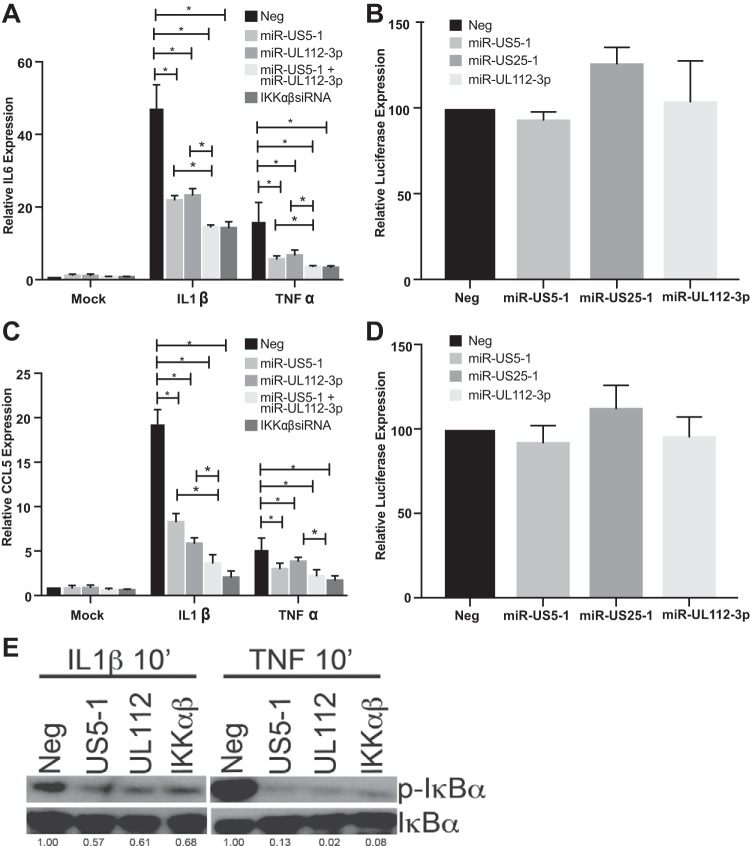
HCMV miR-US5-1 and miR-UL112-3p reduce proinflammatory cytokine transcripts in response to IL-1β and TNF-α. (A) HeLa cells were transfected with negative control (Neg) or double-stranded miR-US5-1 and miR-UL112-3p mimics alone or in combination or with a combination of siRNAs targeting the IKKα and IKKβ transcripts. Forty-eight hours after transfection, cells were treated with IL-1β or TNF-α for 16 h and then RNA was harvested for quantitative RT-PCR using primer/probe sets for IL-6. (B) A dual luciferase reporter containing the IL-6 3′ UTR was transfected into 293T cells along with negative control or double-stranded HCMV miRNA mimics. Twenty-four hours after transfection, luciferase expression was assayed. (C) The experiment was performed as in panel A using a CCL5 primer/probe set. (D) The experiment was performed as in panel B using a dual luciferase vector containing the CCL5 3′ UTR. (E) HeLa cells were transfected with negative control, miR-US5-1 or miR-UL112-3p double-stranded mimics, or a combination of siRNAs targeting the IKKα and IKKβ transcripts for 48 h and then treated with IL-1β or TNF-α for 10 min. Lysates were harvested and blotted for phospho- and total IκBα. Relative band intensity was determined by dividing the intensity of the band by GAPDH followed by normalization to the negative control and presented numerically beneath each lane. *, *P*  < 0.05.

HCMV is known to block NF-κB signaling at late times postinfection (17–19), although the gene product(s) involved in this process has not been fully elucidated. Given the early-late expression kinetics of the HCMV miRNAs, we asked whether viral miRNAs play a role in interfering with NF-κB signaling. To this end, we transfected HeLa cells with negative-control miRNA; HCMV miRNAs, alone or in combination; or small interfering RNAs (siRNAs) targeting IKKα and IKKβ and assessed IL-6 and CCL5 transcript levels after stimulation with IL-1β or TNF-α. We determined that HCMV miRNAs miR-US5-1 and miR-UL112-3p were capable of significantly reducing IL-6 and CCL5 transcript levels in response to both IL-1β and TNF-α. Combining the miRNAs had a significantly enhanced effect on IL-6 and CCL5 transcript levels compared to that with each miRNA alone, except for the effect of miR-US5-1 on CCL5 levels in TNF-α-treated cells, and levels were similar to those observed in cells transfected with siRNAs targeting IKKα and IKKβ (Fig. 1A and C). Importantly, neither miR-US5-1, miR-UL112-3p, nor the unrelated HCMV miRNA miR-US25-1 directly targets the 3′ UTR of IL-6 or CCL5, as determined by luciferase assay (Fig. 1B and D). These data suggest that the HCMV miRNAs function to limit proinflammatory cytokine production through targeting upstream components of the NF-κB signaling pathway.

In order to determine where in the NF-κB signaling pathway the miRNAs exert their effect, we first analyzed the phosphorylation status of IκBα in the presence of the miRNAs. The IKK complex phosphorylates IκBα in response to upstream signals, leading to its proteasome-dependent degradation. We assessed whether miR-US5-1 and miR-UL112-3p could affect the phosphorylation of IκBα in response to IL-1β and TNF-α treatment. HeLa cells were transfected with negative-control miRNA, miR-US5-1, miR-UL112-3p, or a combination of siRNAs targeting IKKα and IKKβ. Forty-eight hours after transfection, cells were treated with IL-1β or TNF-α for 10 min and then protein lysates were harvested and immunoblotted for total and phospho-IκBα. Exogenous expression of miR-US5-1 and miR-UL112-3p resulted in decreased IκBα phosphorylation in response to either IL-1β or TNF-α, similar to the results using siRNAs targeting IKKα and IKKβ (Fig. 1E). Thus, the HCMV miRNAs are likely targeting proteins in the NF-κB signaling pathway upstream of IκBα phosphorylation.

### IKKα and IKKβ are targets of the HCMV miRNAs miR-US5-1 and miR-UL112-3p.

**FIG 2:**
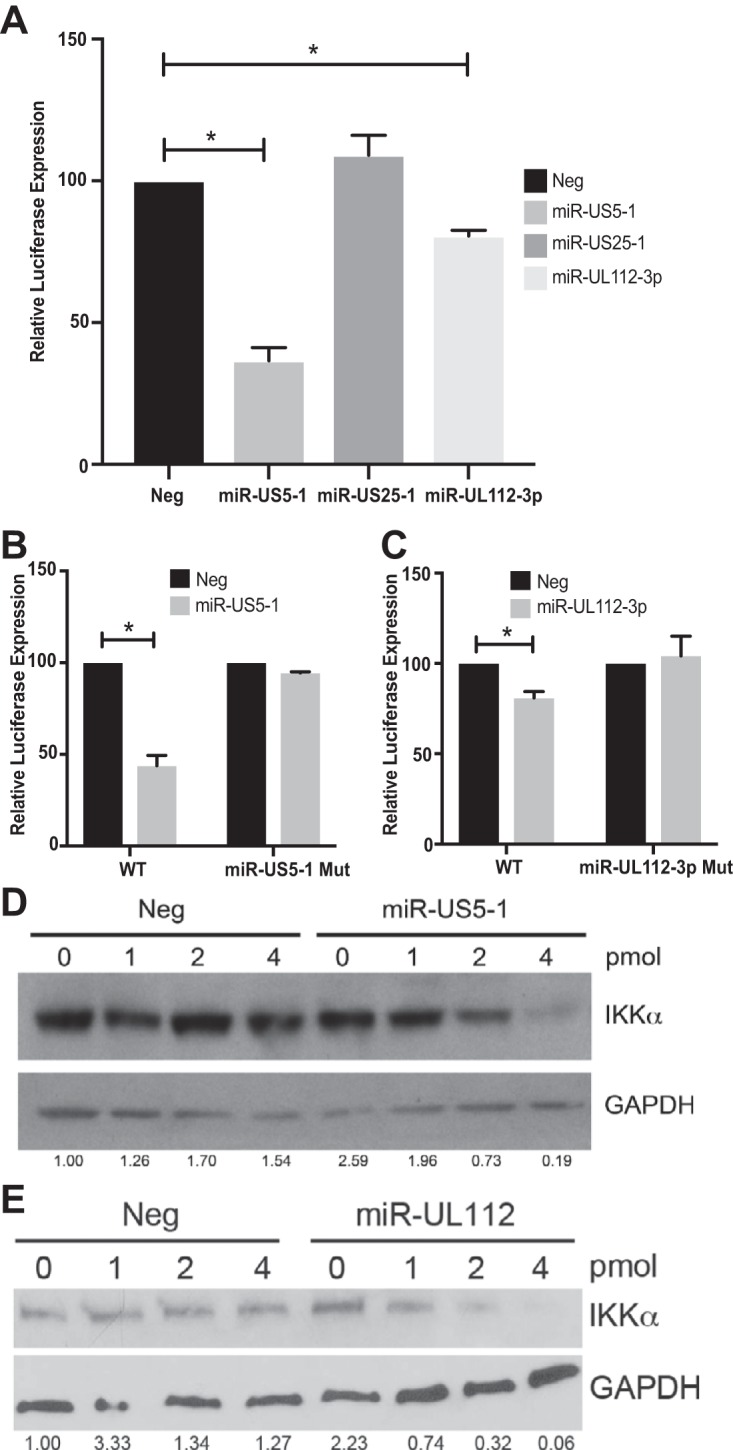
miR-US5-1 and miR-UL112-3p directly target IKKα through sites within the 3′ UTR. (A) A dual luciferase reporter containing the 3′ UTR of IKKα was transfected into 293T cells along with negative control (Neg) or double-stranded HCMV miRNA mimics. Luciferase expression was assessed 24 h posttransfection. (B) Luciferase assays were performed as in panel A using dual luciferase vectors containing either WT IKKα 3′ UTR or a mutation in the putative miR-US5-1 binding site. (C) Luciferase assays were performed as in panel A using dual luciferase vectors containing either WT IKKα 3′ UTR or a mutation in the putative miR-UL112-3p binding site. (D) 293T cells were transfected with increasing amounts of negative control or miR-US5-1 double-stranded mimic for 48 h, after which lysates were harvested and blotted for IKKα and GAPDH. (E) 293T cells were transfected with increasing amounts of negative control or miR-UL112-3p mimic for 48 h, after which lysates were harvested and blotted for IKKα and GAPDH. Relative band intensity was determined by dividing the intensity of the band by GAPDH followed by normalization to the “0” negative control and presented numerically beneath each lane. *, *P* < 0.05.

**FIG 3:**
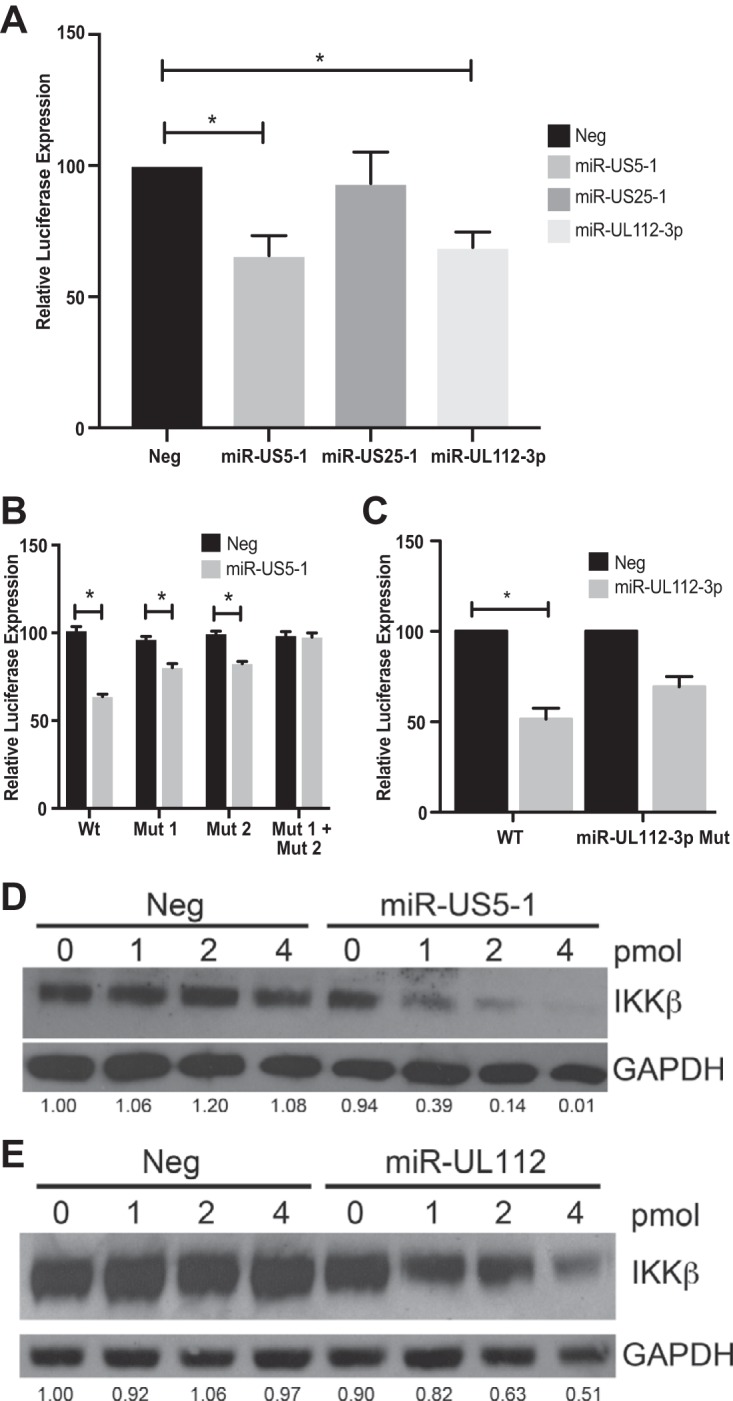
miR-US5-1 and miR-UL112-3p directly target IKKβ through sites within the 3′ UTR. (A) A dual luciferase reporter containing the 3′ UTR of IKKβ was transfected into 293T cells along with negative control (Neg) or double-stranded HCMV miRNA mimics. Luciferase expression was assessed 24 h posttransfection. (B) Luciferase assays were performed as in panel A using dual luciferase vectors containing either WT IKKβ 3′ UTR or mutations in the putative miR-US5-1 binding sites alone or in combination. (C) Luciferase assays were performed as in panel A using dual luciferase vectors containing either WT IKKβ 3′ UTR or a mutation in the putative miR-UL112-3p binding site. (D) 293T cells were transfected with increasing amounts of negative control or miR-US5-1 double-stranded mimic for 48 h, after which lysates were harvested and blotted for IKKβ and GAPDH. (E) 293T cells were transfected with increasing amounts of negative control or miR-UL112-3p double-stranded mimic for 48 h, after which lysates were harvested and blotted for IKKβ and GAPDH. Relative band intensity was determined by dividing the intensity of the band by GAPDH followed by normalization to the “0” negative control and presented numerically beneath each lane. *, *P* < 0.05.

Bioinformatic analysis was used to identify genes within the IL-1β and TNF-α arms of the NF-κB pathway that could be potential targets for HCMV miRNAs. Given that miR-US5-1 and miR-UL112-3p were able to block NF-κB signaling in response to both IL-1β and TNF-α, we focused on potential targets at the point of convergence of these pathways: the IKK complex composed of the kinases IKKα and IKKβ and the scaffolding protein IKKγ or NEMO. By analyzing the 3′ UTRs of each gene, we determined that IKKα and IKKβ contain potential miRNA binding sites for both miR-US5-1 and miR-UL112-3p. We tested the ability of HCMV miRNAs to target the 3′ UTRs of IKKα and IKKβ using dual luciferase reporters. miR-US5-1 and miR-UL112-3p significantly reduced luciferase expression in constructs containing the IKKα (Fig. 2A) and IKKβ (Fig. 3A) 3′ UTRs compared to negative-control miRNA, whereas a third HCMV miRNA, miR-US25-1, did not affect luciferase expression from these constructs. We next identified the miR-US5-1 and miR-UL112 binding sites within the IKKα and IKKβ 3′ UTRs by altering the predicted regions using site-directed mutagenesis. Our bioinformatics analysis identified a miR-US5-1 6-mer 789 nucleotides downstream of the IKKα stop codon. Removal of these 6 nucleotides restored luciferase levels in cells transfected with miR-US5-1 to those seen with negative-control miRNA transfection (Fig. 2B). Additionally, we identified one miR-UL112-3p 7-mer beginning 4 nucleotides downstream of the stop codon of IKKα. Removal of these 7 nucleotides resulted in luciferase levels in cells transfected with miR-UL112-3p similar to those in negative-control-transfected cells (Fig. 2C). Using site-directed mutagenesis, we determined that the IKKβ 3′ UTR contains one miR-US5-1 6-mer (starting at nucleotide 258 of the 3′ UTR) and one noncanonical miR-US5-1 site from nucleotides 184 to 208. Mutation of both of these miR-US5-1 binding sites was required to restore luciferase expression to levels observed with upon transfection with a negative-control mimic (Fig. 3B). In addition, mutation of a noncanonical miR-UL112-3p site at nucleotides 764 to 783 in the 3′ UTR of IKKβ resulted in luciferase expression similar to that in negative-control-transfected cells (Fig. 3C).

We next assessed the ability of miR-US5-1 and miR-UL112-3p mimics to downregulate IKKα and IKKβ protein levels in transfected cells. 293T cells were transfected with increasing amounts of negative-control miRNA, miR-US5-1, or miR-UL112-3p mimic. Seventy-two hours after transfection, protein lysates were harvested and blotted for IKKα or IKKβ. As shown in Fig. 2D and E and 3D and E, both miR-US5-1 and miR-UL112-3p were able to decrease IKKα and IKKβ protein levels in a dose-dependent manner. These data suggest that the partial block in NF-κB signaling observed in the presence of these miRNAs is due, at least in part, to limiting expression of the kinases available to phosphorylate IκBα.

### HCMV miRNAs target IKKα and IKKβ during infection.

**FIG 4:**
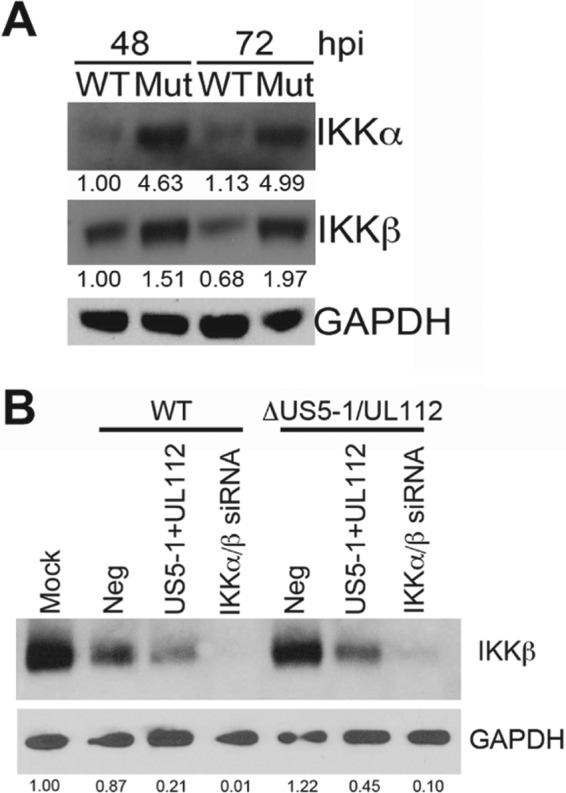
IKKα and IKKβ protein levels are increased during infection with a miR-US5-1/miR-UL112-3p double mutant virus. (A) NHDF were infected with WT and miR-US5-1/miR-UL112-3p double mutant (Mut) virus for 48 and 72 h, after which lysates were harvested and immunoblotted for IKKα, IKKβ, and GAPDH. (B) NHDF were transfected with negative control (Neg), miR-US5-1 and miR-UL112-3p double-stranded mimics, or siRNAs targeting the IKKα and IKKβ transcripts. Forty-eight hours after transfection, cells were infected with WT or miR-US5-1/miR-UL112-3p double mutant viruses for 72 h and then protein lysates were harvested and immunoblotted for IKKβ and GAPDH. Relative band intensity was determined by dividing the intensity of the band by GAPDH followed by normalization to the 48-h WT sample (in panel A) or mock (in panel B) and presented numerically beneath each lane.

In order to determine the functional role for HCMV miRNA targeting of IKKα and IKKβ during infection, we derived HCMVs that carry mutations in miR-UL112-3p that disrupt miRNA expression while maintaining the open reading frame sequence of UL114 (37) and in which the miR-US5-1 pre-miRNA sequence was removed (42). This virus replicates with WT kinetics in single-step growth curves and expresses viral proteins and miRNAs with similar kinetics (42) (data not shown). Normal human dermal fibroblasts (NHDF) were infected with WT and miR-US5-1/miR-UL112-3p double mutant virus at a multiplicity of infection (MOI) of 3, and protein lysates were harvested at 48 and 72 h postinfection (hpi). As demonstrated in Fig. 4A, infection with the miR-US5-1/miR-UL112-3p double mutant virus results in increased accumulation of IKKα and IKKβ proteins compared to cells infected with WT virus. To confirm that the increase in IKKα and IKKβ levels is due to the lack of miR-US5-1 and miR-UL112-3p expression, NHDF were transfected with negative-control miRNA, miR-US5-1 and miR-UL112-3p mimics, or siRNAs targeting the IKKα and IKKβ transcripts. Forty-eight hours after transfection, cells were infected with WT or miR-US5-1/miR-UL112-3p double mutant virus for 72 h, and then protein lysates were harvested and immunoblotting for IKKβ and glyceraldehyde-3-phosphate dehydrogenase (GAPDH) was performed. The increase in IKKβ protein levels observed in cells infected with the miR-US5-1/miR-UL112-3p double mutant (compare “Neg” lanes) can be reduced by preexpression of miR-US5-1 and miR-UL112-3p or siRNAs targeting IKKα and IKKβ (Fig. 4B), suggesting that miR-US5-1 and miR-UL112-3p target IKKα and IKKβ during infection.

### HCMV miRNAs are involved in the late block in NF-κB signaling observed in infected fibroblasts.

**FIG 5:**
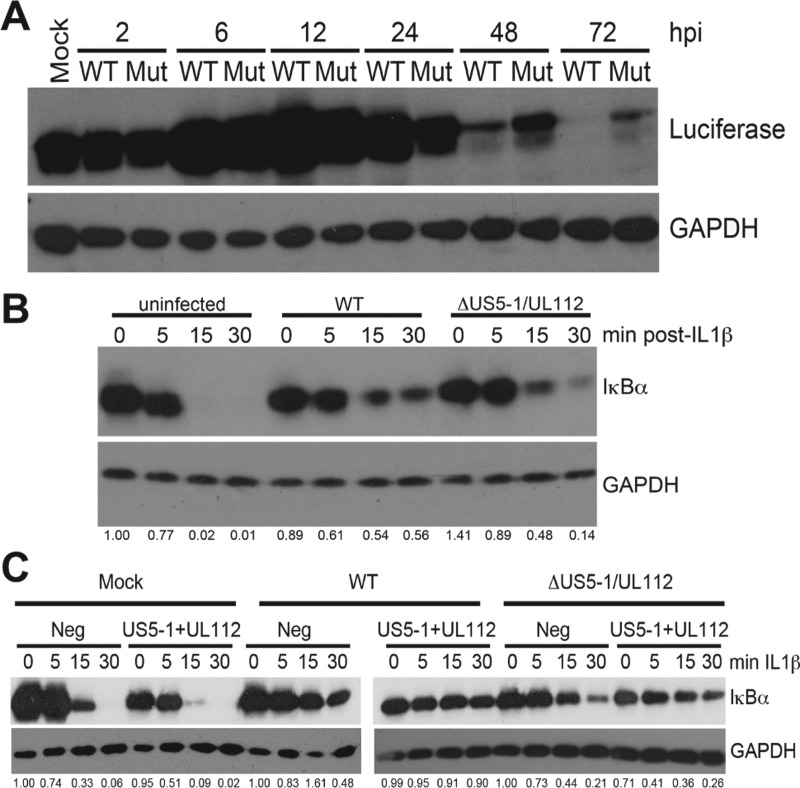
miR-US5-1 and miR-UL112-3p are involved in the late block to NF-κB signaling observed in HCMV-infected fibroblasts. (A) Human fibroblasts expressing a luciferase reporter under the control of the minimal NF-κB promoter were infected with WT or miR-US5-1/miR-UL112-3p double mutant virus (Mut), and protein lysates were harvested at the indicated times. Lysates were immunoblotted for luciferase and GAPDH. (B) NHDF were mock infected or infected with WT or miR-US5-1/miR-UL112-3p double mutant virus for 72 h. After this time, cells were treated with IL-1β, and protein lysates were harvested at the indicated times and immunoblotted for IκBα or GAPDH. Relative band intensity was determined by dividing the intensity of the band by GAPDH followed by normalization to the uninfected, untreated sample and presented numerically beneath each lane. (C) The experiment was performed as in panel B with the exception that the NHDF were first transfected with negative control or miR-US5-1 and miR-UL112-3p mimics for 48 h prior to infection. Relative band intensity was determined by dividing the intensity of the band by GAPDH followed by normalization to each negative-control, untreated sample and presented numerically beneath each lane.

We have previously determined that infection of fibroblasts with WT virus results in a block in NF-κB signaling at late times postinfection (18). Given that miR-US5-1 and miR-UL112-3p play a role in modulating the NF-κB response through targeting IKKα and IKKβ, we asked whether NF-κB signaling initiated by viral infection is altered in cells infected with the miR-US5-1/miR-UL112-3p double mutant virus. To this end, human fibroblasts expressing the luciferase gene under the control of the minimal NF-κB promoter (tHF-NF-κB) were infected with WT and miR-US5-1/miR-UL112-3p double mutant virus, and protein lysates were harvested at various times postinfection and blotted for luciferase protein as a marker of NF-κB activation. As can be seen in Fig. 5A, NF-κB signaling is upregulated by 6 h after infection with both WT and the miR-US5-1/miR-UL112-3p double mutant virus. At 48 to 72 h, a block in NF-κB signaling becomes evident in cells infected with WT virus, as demonstrated by a reduction in luciferase protein accumulation to nearly undetectable levels. Infection with the miR-US5-1/miR-UL112-3p double mutant virus, however, results in prolonged NF-κB signaling compared to WT-infected cells, directly implicating a role for the viral miRNAs in blocking NF-κB signaling.

Another measure of the blockade to NF-κB signaling that occurs at late times in HCMV-infected fibroblasts is the prevention of IκBα degradation in response to exogenous stimuli (18). In uninfected cells, treatment with IL-1β results in the rapid degradation of IκBα. When cells are infected with WT HCMV for 72 h prior to treatment with IL-1β, the degradation of IκBα is blocked (Fig. 5B). However, if the cells are infected with the miR-US5-1/miR-UL112-3p double mutant virus, we have consistently observed only a partial block to IκBα degradation, which can be characterized as an intermediate phenotype between uninfected and WT-infected cells. If miR-US5-1 and miR-UL112-3p are provided in *trans*, through prior transfection with miRNA mimics, the block to IκBα degradation is partially restored (Fig. 5C). These data support the hypothesis that miR-US5-1 and miR-UL112-3p are involved in functionally blocking the NF-κB signaling pathway at late times of infection.

### miR-US5-1 and miR-UL112-3p contribute to blocking NF-κB-responsive cytokine transcript accumulation during viral infection.

**FIG 6:**
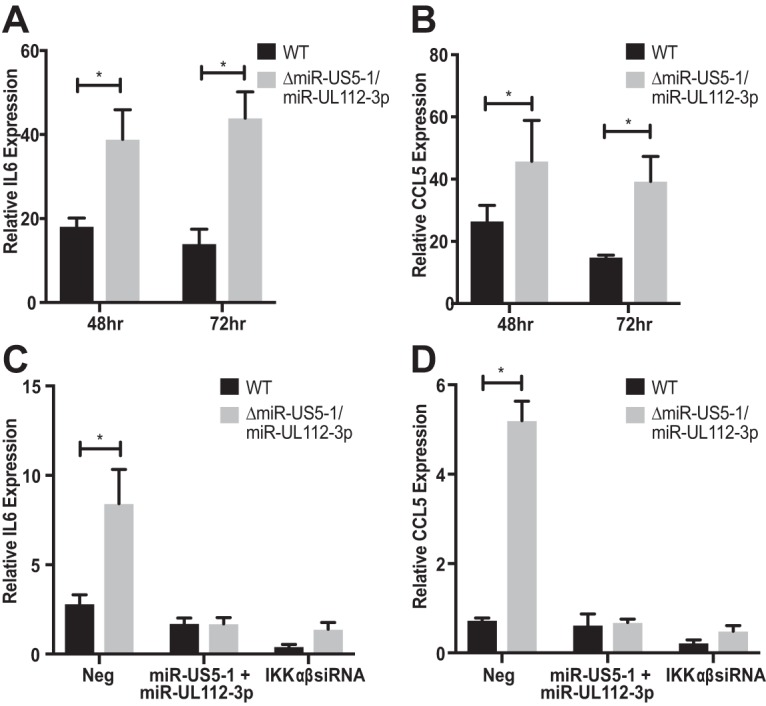
Proinflammatory cytokine transcript levels are elevated during infection with the miR-US5-1/miR-UL112-3p double mutant virus. (A) NHDF were infected with WT and miR-US5-1/miR-UL112-3p double mutant virus, and then RNA was harvested at 48 and 72 h postinfection and subjected to quantitative RT-PCR for IL-6. (B) The experiment was performed as in panel A, analyzing CCL5 transcript levels. (C) NHDF were transfected with negative control (Neg), miR-US5-1 and miR-UL112-3p double-stranded mimics, or siRNAs targeting the IKKα and IKKβ transcripts. Forty-eight hours posttransfection, the cells were infected with WT or miR-US5-1/miR-UL112-3p double mutant virus. RNA was harvested 72 h postinfection and analyzed for IL-6 transcript levels using quantitative RT-PCR. (D) The experiment was performed as in panel C, analyzing CCL5 transcript levels. *, *P* < 0.05.

Through partially blocking the phosphorylation and degradation of IκBα, miR-US5-1 and miR-UL112-3p may affect the downstream expression of proinflammatory cytokines whose transcription depends on binding of the NF-κB subunits. To test this, NHDF were infected with WT and miR-US5-1/miR-UL112-3p double mutant virus for 48 and 72 h, after which time RNA was harvested and analyzed for IL-6 and CCL5 transcript accumulation. As seen in Fig. 6A (IL-6) and B (CCL5), cytokine transcript levels are higher in cells infected with the miRNA mutant virus than in WT-infected cells. To determine if the increased transcript levels observed in miRNA mutant virus-infected cells could be restored by preexpression of the miRNAs, NHDF were transfected with negative-control miRNA, miR-US5-1, and miR-UL112-3p or siRNAs targeting the IKKα and IKKβ transcripts and then 48 h later infected with WT or the miR-US5-1/miR-UL112-3p double mutant virus. As seen in Fig. 6C and D, infection with the miR-US5-1/miR-UL112-3p mutant virus resulted in increased accumulation of IL-6 and CCL5 transcripts, respectively, compared to WT-infected cells in the presence of negative-control miRNA; however, preexpression of miR-US5-1 and miR-UL112-3p was able to restore cytokine transcript accumulation in miRNA mutant virus-infected cells to levels observed during WT infection and similar to those observed in infection in the presence of siRNAs targeting IKKα and IKKβ.

**FIG 7:**
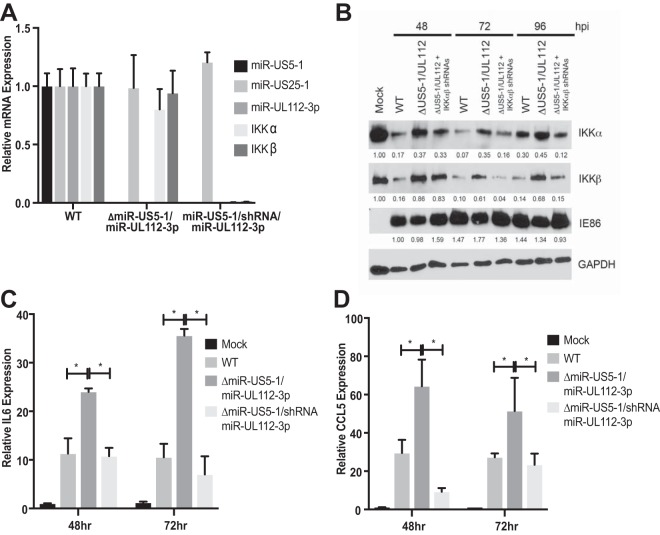
Expression of IKKα and IKKβ shRNAs in the context of the miR-US5-1/miR-UL112-3p double mutant virus complements the defect in NF-κB signaling. (A) NHDF were infected with WT, miR-US5-1/miR-UL112-3p double mutant, or miR-US5-1/shRNA/miR-UL112-3p virus (shRNA hairpins targeting the IKKα and IKKβ transcripts in place of the miR-US5-1 hairpin). Seventy-two hours after infection, RNA was harvested and quantitative RT-PCR for HCMV miR-US5-1, miR-US25-1, miR-UL112-3p, IKKα, or IKKβ was performed. Expression levels were normalized to miR-16 and compared to WT-infected cells. (B) NHDF were infected with WT and miR-US5-1/miR-UL112-3p and miR-US5-1/shRNA/miR-UL112-3p mutant viruses, and protein was harvested at the indicated times and immunoblotted for IKKα, IKKβ, HCMV IE86, and GAPDH. Relative band intensity was determined by dividing the intensity of the band by GAPDH followed by normalization to mock (or 48-h WT in the case of IE86) sample and presented numerically beneath each lane. (C) NHDF were infected as in panel B, and RNA was harvested at 48 and 72 h postinfection. Quantitative RT-PCR was performed using primer/probe sets for IL-6 and U6 (as a normalization control). (D) The experiment was performed as in panel C using a primer/probe set for CCL5. *, *P* < 0.05.

While these data strongly suggest that miR-US5-1 and miR-UL112-3p affect cytokine transcript accumulation due to targeting IKKα and IKKβ, it remains possible that the phenotype is partially due to affecting expression of additional, known or unknown targets of the miRNAs. In order to directly link the phenotypes observed with the miR-US5-1/miR-UL112-3p mutant virus with its effects on targeting IKKα and IKKβ, we derived a mutant virus where hairpins expressing IKKα and IKKβ shRNAs were inserted in place of the miR-US5-1 hairpin in the background of a miR-UL112-3p mutant (miR-US5-1/shRNA/miR-UL112-3p). As shown in Fig. 7A, the resultant virus does not express miR-US5-1 or miR-UL112-3p but does result in decreased IKKα and IKKβ mRNA levels compared to WT-infected cells. Infection of NHDF with the miRNA mutant virus expressing the IKKα and IKKβ shRNAs results in IKKα and IKKβ protein levels similar to those observed in cells infected with the WT virus and not the parent miRNA mutant virus by 72 h postinfection (hpi), while IE86 levels remained similar for all viruses (Fig. 7B). Transcript levels for IL-6 (Fig. 7C) and CCL5 (Fig. 7D) are reduced in cells infected with the shRNA-expressing virus compared to the parent miR-US5-1/miR-UL112-3p mutant virus, strongly suggesting that the lack of miRNA targeting of IKKα and IKKβ is responsible for the increase in cytokine transcript levels observed during infection with the miRNA mutant virus.

### Targeting of IKKα and IKKβ by miR-US5-1 and miR-UL112-3p results in increased secretion of proinflammatory cytokines from multiple cell types.

**FIG 8:**
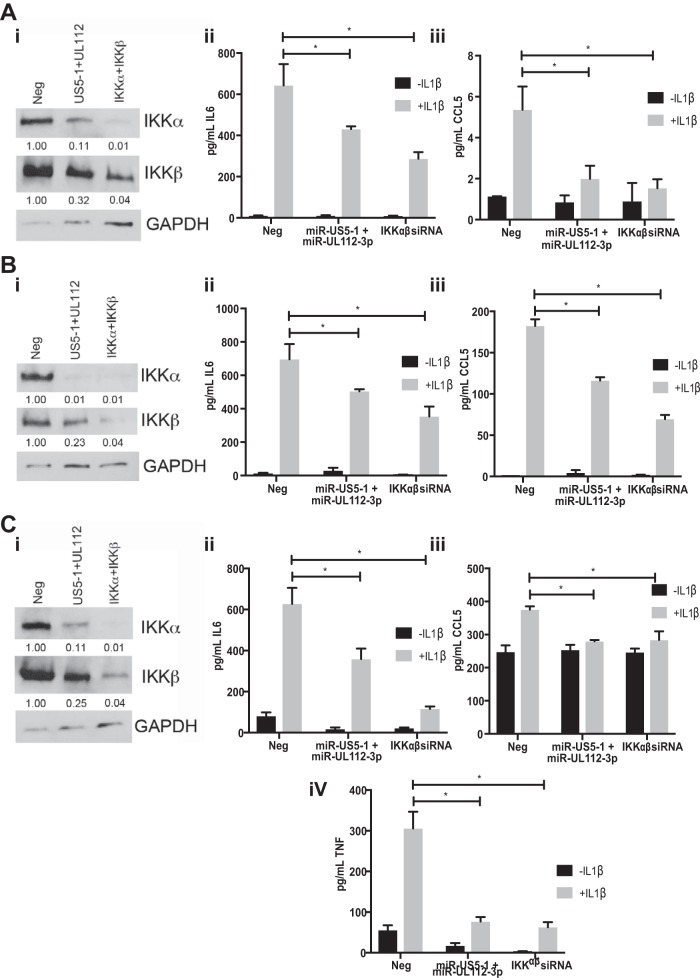
miR-US5-1 and miR-UL112-3p alone block proinflammatory cytokine production in cell types relevant to HCMV infection. (A) NHDF. (B) hAEC. (C) PMA-treated THP-1 cells. Cells were transfected with negative control (Neg), miR-US5-1 and miR-UL112-3p double-stranded mimics, or siRNAs targeting the IKKα and IKKβ transcripts. (i) Protein lysates were harvested 72 h posttransfection and immunoblotted for IKKα, IKKβ, and GAPDH. Relative band intensity was determined by dividing the intensity of the band by GAPDH followed by normalization to the negative-control sample and presented numerically beneath each lane. (ii) Cells were transfected for 72 h and then treated with IL-1β for 8 h. Supernatants were harvested and analyzed for IL-6 protein levels. (iii) Supernatants were harvested and analyzed for CCL5 protein levels. (iv) TNF-α levels were assessed in transfected THP-1 cells. *, *P* < 0.05.

We next assessed whether miR-US5-1 and miR-UL112-3p alone could affect cytokine production in cell types relevant to HCMV infection *in vivo*. To this end, we transfected NHDF, human aortic endothelial cells (hAEC), and the monomyelocytic cell line THP-1 with negative-control miRNA, miR-US5-1 and miR-UL112-3p, or siRNAs targeting IKKα and IKKβ for 72 h and then assessed protein levels in cell lysates and cytokine levels in the supernatant 8 h after treatment with IL-1β. As shown in Fig. 8A, B, and C (panels i), cells transfected with miR-US5-1 and miR-UL112-3p mimics or siRNAs targeting IKKα and IKKβ showed reduced IKKα and IKKβ protein levels compared to negative-control-transfected cells. Additionally, expression of miR-US5-1 and miR-UL112-3p, as well as siRNAs targeting IKKα and IKKβ, reduced IL-6 (panels ii), CCL5 (panels iii), and TNF-α (in THP-1) (panel iv) levels in the supernatant compared to negative-control-transfected cells. Interestingly, THP-1 cell cultures had higher basal levels of IL-6 and CCL5 in the supernatants than did NHDF and hAEC, likely due to the fact that the cells were stimulated to differentiate with phorbol myristate acetate (PMA) prior to transfection. However, these levels were still reduced upon introduction of miRNA mimics or siRNAs targeting IKKα and IKKβ.

**FIG 9:**
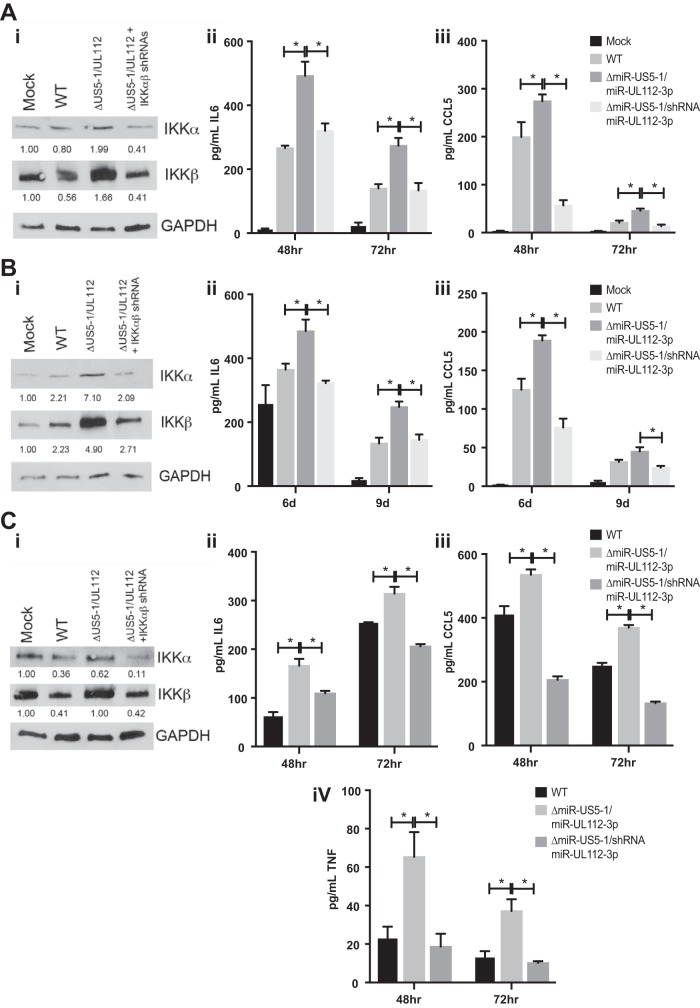
miR-US5-1 and miR-UL112-3p block proinflammatory cytokine production during HCMV infection. (A) NHDF. (B) hAEC. (C) PMA-treated THP-1 cells. Cells were infected with WT or miR-US5-1/miR-UL112-3p or miR-US5-1/shRNA/miR-UL112-3p mutant viruses. (i) Protein lysates were harvested 72 h postinfection and immunoblotted for IKKα, IKKβ, and GAPDH. Relative band intensity was determined by dividing the intensity of the band by GAPDH followed by normalization to the negative-control sample and presented numerically beneath each lane. (ii) Supernatants were harvested at 48 and 72 h postinfection and analyzed for IL-6 protein levels. (iii) Supernatants were harvested and analyzed for CCL5 protein levels. (iv) TNF-α levels were assessed in infected THP-1 cells. *, *P* < 0.05.

We next assessed whether the increased cytokine transcript levels that we detected upon infection with the miR-US5-1/miR-UL112-3p mutant virus resulted in increased production of proinflammatory cytokines in NHDF, hAEC, and THP-1 cells. To this end, we infected NHDF, hAEC, and THP-1 cells with WT and miR-US5-1/miR-UL112-3p and miR-US5-1/shRNA/miR-UL112-3p mutant viruses and harvested protein at 72 hpi (NHDF and THP-1 cells) or 9 dpi (hAEC) and supernatants at the indicated time points (Fig. 9A, B, and C). As expected, there were increased levels of IKKα and IKKβ protein in cells infected with the miR-US5-1/miR-UL112-3p double mutant virus compared to WT-infected cells that were reduced upon infection with the miRNA mutant expressing shRNAs targeting IKKα and IKKβ in all cell types tested (panels i). The miR-US5-1/miR-UL112-3p mutant virus infection resulted in increased secretion of IL-6 (panels ii), CCL5 (panels iii), and TNF-α (in THP-1 cells) (panel iv) compared to WT-infected cells, which was significantly reduced in cells infected with the miRNA mutant virus expressing shRNAs targeting IKKα and IKKβ. WT infection of PMA-treated THP-1 cells resulted in higher levels of CCL5 in the supernatant than did infection of NHDF or hAEC and higher levels of IL-6 than did infection of NHDF. However, infection with the miR-US5-1/miR-UL112-3p mutant virus still enhanced IL-6 and CCL5 levels above that observed with WT infection. Together, these data demonstrate that the functional consequence of prolonged NF-κB signaling in the absence of miR-US5-1 and miR-UL112-3p is increased expression and secretion of proinflammatory cytokines in cell types relevant to HCMV infection *in vivo*.

## DISCUSSION

In the current study, we demonstrate that the late block to NF-κB signaling observed in HCMV-infected fibroblasts is due in part to the action of the viral miRNAs miR-US5-1 and miR-UL112-3p. By reducing expression of both IKKα and IKKβ, the HCMV miRNAs participate in preventing IκBα phosphorylation and degradation, thereby preventing the release of the NF-κB subunits and reducing proinflammatory cytokine expression in cell types relevant to HCMV persistence and latency *in vivo*, including fibroblasts, endothelial cells, and monocytes.

By targeting proteins at the point of convergence of numerous innate signaling pathways, HCMV miRNAs can modulate signaling by multiple NF-κB-inducing factors using minimal genetic material. It appears that HCMV miRNAs target and disable signaling hubs in order to impair cellular signaling pathways. This may be especially important in the case of latency, where only a subset of HCMV miRNAs (43 and our unpublished observations) and few viral gene products are produced but the cell must be maintained in a quiescent state. Although the effect of a single miRNA on a single transcript may be subtle, multiple miRNAs targeting multiple components of a cellular signaling pathway can have significant effects. We have previously shown that three HCMV miRNAs act in concert to target multiple components of the cellular endocytic recycling pathway to affect formation of the virion assembly compartment (42). In this case, the full phenotype is apparent only when all three miRNAs are present, demonstrating that subtle effects on individual protein levels can be amplified when multiple proteins are targeted. Likewise, we show here that by utilizing two miRNAs to target different components of the IKK complex, the virus significantly dampens the effects of activating NF-κB signaling pathways using different exogenous stimuli. Another method to maximize the effects of viral miRNAs on protein expression is to have multiple miRNAs target the same transcript (46). We show here that two miRNAs target both IKKα and IKKβ, allowing for maximal downregulation of protein expression. It is possible that in different cell types or stages of the viral life cycle, the expression levels of miR-US5-1 and miR-UL112-3p differ, thus requiring multiple miRNA target sites to ensure protein downregulation. For example, we have shown that miR-UL112-3p is expressed with higher relative abundance in hAEC than in fibroblasts and THP-1 cells (unpublished observations). The data presented in Fig. 2 and 3 suggest that IKKα and IKKβ are affected by miR-US5-1 and miR-UL112-3p to differing extents. As such, the ratios of IKKα and IKKβ could be altered in different infected cell types, which could have important functional consequences. Canonical NF-κB signaling utilizes an IKK complex composed of IKKα, IKKβ, and IKKγ in order to phosphorylate IκBα and release the p50 and p65 (RelA) subunits, whereas noncanonical NF-κB signaling uses IKKα homodimers to phosphorylate p105 and release the p52 and RelB subunits. By differentially altering the ratios of IKKα and IKKβ in different cell types, the virus may be not only restricting but specifically modulating the release of NF-κB subunits to suit its purposes. Although not directly investigated in this study, the effects of the viral miRNAs on IKKα and IKKβ protein levels could also alter the degradation of alternative IκB proteins, including IκBβ and IκBε, which would also affect the release of NF-κB subunits.

The data presented here suggest that while miR-US5-1 and miR-UL112-3p play important roles in modulating NF-κB signaling at late times of infection, other viral gene products are also involved. As demonstrated in Fig. 5A, NF-κB signaling is reduced from its peak at 12 to 24 hpi in both WT- and miRNA mutant virus-infected cells. Additionally, we have demonstrated an intermediate phenotype, whereby there is only a partial block to IκBα degradation in response to IL-1β, in cells infected with the miRNA mutant virus compared to WT-infected cells (Fig. 5B). To date, no other viral gene product has been identified which participates in the late block to NF-κB signaling, although the tegument protein UL26 was recently demonstrated to block TNF-α-mediated IKK phosphorylation (20). However, previous work suggests that a viral protein with late kinetics is required to prevent IL-1β- and TNF-α-mediated IκBα degradation (18).

Through the use of a short region of complementarity, a single miRNA can potentially target hundreds of different cellular transcripts. While preexpression of miR-US5-1 and miR-UL112-3p can at least partially restore the phenotype of the miRNA mutant virus observed in Fig. 5 and 6, this does not directly demonstrate that the observed effects of the exogenously expressed miRNAs are due specifically to targeting of IKKα and IKKβ directly. Our group and others have identified additional cellular and viral targets of miR-US5-1 and miR-UL112-3p (38, 40, 42, 46–50), including components within the TLR2 signaling pathway upstream of IKK complex activation (45). Because of the potentially pleiotropic effects of preventing expression of a viral miRNA in the context of viral infection, we wanted to determine whether the increase in cytokine expression observed with a miR-US5-1/miR-UL112-3p double mutant virus was due specifically to the miRNAs targeting IKKα and IKKβ. To address this, we designed a viral mutant whereby the hairpin for miR-US5-1 was replaced with an shRNA expression cassette containing hairpins targeting IKKα and IKKβ in the context of a miR-UL112-3p mutant. The rationale for this design was, first, to allow for depletion of IKKα and IKKβ transcripts only in cells that were infected with HCMV and, second, to ensure that this happens with kinetics similar to expression of the viral miRNAs. In fibroblasts, miRNA expression is detected at low levels early after infection, with miRNA levels increasing throughout the lytic life cycle. As shown in Fig. 7B, levels of IKKα and IKKβ are decreased at 48 hpi in cells infected with the miR-US5-1/miR-UL112-3p mutant virus expressing the IKKα and IKKβ shRNAs compared to the parental mutant virus and reach levels similar to WT virus by 72 hpi. While this miRNA mutant virus recapitulates WT levels of IKKα and IKKβ in infected cells, it also reduces proinflammatory cytokine levels to those observed during WT infection (Fig. 9), indicating that miR-US5-1 and miR-UL112-3p targeting of IKKα and IKKβ, and not other targets of these miRNAs, is primarily responsible for the phenotype.

By interfering with proinflammatory cytokine production, miR-US5-1 and miR-UL112-3p participate in key aspects of the viral life cycle in numerous cell types. In fibroblasts and endothelial cells, limiting proinflammatory cytokine production is likely necessary to limit the recruitment of immune cells and killing of the infected cell as well as for preventing apoptosis (51). During latency in myeloid cells, proinflammatory cytokine signaling leads to cellular differentiation and reactivation of lytic viral replication. In this case, the role of miRNA modulation of NF-κB signaling could be 2-fold. First, in cases of suboptimal stimuli, the miRNAs may act to prevent any spurious activation of the NF-κB signaling pathway that could lead to abortive or incomplete differentiation and viral reactivation. Second, once an optimal reactivation stimulus has occurred and reactivation/replication of the virus is ongoing, the miRNAs may again help to limit the negative effects of proinflammatory cytokine production in the resultant macrophages.

HCMV evades innate and adaptive immune responses using a remarkable array of proteins. It is now appreciated that the noncoding RNAs of HCMV also play essential roles in all aspects of the HCMV life cycle, including manipulating many cellular signaling pathways. HCMV encodes IE86 (21) and UL26 (20), which act to block NF-κB subunit binding and IKKα phosphorylation, respectively. Here, we show that HCMV-encoded miRNAs additionally manipulate NF-κB signaling by directly reducing the expression of components of the IKK complex. Previous work has demonstrated that HCMV miRNAs manipulate cellular pathways that affect proinflammatory cytokine production and release by additional means, including targeting proteins involved in activation of TLR2 signaling (45), the endocytic recycling pathway (42), and activin A signaling (43) and targeting CCL5/RANTES directly (44). The use of both proteins and miRNAs to alter the effects of NF-κB signaling reflects the diverse features of the HCMV life cycle and is also observed during gammaherpesvirus infection (34–36, 52, 53). HCMV miRNAs accumulate throughout lytic infection, and could, along with viral proteins, regulate both virally induced NF-κB signaling (9, 10, 14) and NF-κB signaling in situations of inflammation, where external stimuli derived from activated immune cells trigger the pathway. Perhaps most importantly, expression of viral miRNAs during latency may allow for manipulation of host signaling pathways by nonimmunogenic molecules that can help the latently infected cell remain poised for reactivation.

## MATERIALS AND METHODS

### Cells, virus, and reagents.

Normal human dermal fibroblasts (NHDF), HeLa cells, human aortic endothelial cells (hAEC), and THP-1 and 293T cells were obtained from the American Type Culture Collection. NHDF and HeLa and HEK293T cells were maintained in Dulbecco’s modified Eagle’s medium (DMEM) supplemented with 10% fetal bovine serum (FBS; HyClone) and 100 U/ml of penicillin and 100 µg/ml of streptomycin (Invitrogen). hAEC were maintained in endothelial growth medium 2 (EGM-2) with associated supplements excluding heparin, as well as 10% FBS, penicillin, and streptomycin. THP-1 cells were maintained in RPMI with 10% FBS, penicillin, and streptomycin. Human fibroblasts expressing an NF-κB-responsive luciferase construct (tHF-NF-κB) were a kind gift from Victor DeFillipis and maintained as NHDF with 3 µg/ml puromycin. Viruses used in this study include WT strain TRTF or TB40/E and mutant viruses in both strains lacking the pre-miRNA sequence for miR-US5-1 (42) and containing mutations in miR-UL112-3p as in reference 37. Additionally, a virus containing shRNAs targeting IKKα and IKKβ in place of miR-US5-1 in the miR-US5-1/miR-UL112-3p bacterial artificial chromosome (BAC) was constructed using *galK* recombineering. Briefly, the galactokinase (*galK*) gene was used to replace the miR-US5-1 hairpin using homologous recombination. The *galK* gene was replaced with two copies of shRNAs for IKKα and IKKβ with the following sequence: CCGGTAGGGTCTGGGATTCGATATTCTCGAGAATATCGAATCCCAGACCCTATTTTTGTTCAAGAGACCGGGCTGGTTCATATCTTGAACATCTCGAGATGTTCAAGATATGAACCAGCTTTTTCTTCCTGTCACCGGGCAGATGACGTATGGGATATCCTCGAGGATATCCCATACGTCATCTGCTTTTTTGACTGTCCTTCCCGGCCAGCCAAGAAGAGTGAAGAACTCGAGTTCTTCACTCTTCTTGGCTGGTTTTT. NHDF or tHF-NF-κB cells were infected with HCMV at 3 PFU/cell, and hAEC and THP-1 cells were infected with HCMV at 5 PFU/cell for 2 h at 37°C. After this time, the inoculum was removed and replaced with fresh medium and samples were harvested as appropriate for each experiment. IL-1β and TNF-α were obtained from R&D Systems. siRNAs targeting IKKα and IKKβ were obtained from Applied Biosystems.

### Western blotting.

Protein extracts were lysed using 2× SDS lysis buffer (50 mM Tris, pH 6.8, 20% glycerol, 2% SDS), and 2× SDS loading buffer (100 mM Tris, pH 6.8, 20% glycerol, 4% SDS, 0.04% bromophenol blue) was added. Extracts were run on an 8% SDS-PAGE gel, transferred to Immobilon-P transfer membranes (Millipore Corp., Bedford, MA), and visualized with antibodies specific for IKKα (Cell Signaling), IKKβ (Cell Signaling), IκBα (Santa Cruz), phospho-IκBα (Cell Signaling), IE86 (monoclonal antibody [MAb] 810; Millipore), luciferase (Sigma), and GAPDH (Abcam). The relative intensity of bands detected by Western blotting was quantitated using ImageJ software.

### Luciferase assays.

The putative 3′ UTRs of IKKα, IKKβ, IL-6, and CCL5 were cloned into the dual luciferase reporter pSiCheck2 (Clontech). Site-directed mutagenesis was performed using the QuikChange PCR method to mutate the potential miR-US5-1 and miR-UL112-3p binding sites within the IKKβ 3′ UTR, while the miR-US5-1 and miR-UL112-3p binding sites were removed from the IKKα 3′ UTR. HEK293T cells seeded into 96-well plates were cotransfected in triplicate with 100 ng of plasmid and 100 fmol of miRNA mimic (custom designed; IDT) using Lipofectamine 2000 (Invitrogen). Cells were incubated overnight and then harvested for luciferase assay using the Dual-Glo reporter assay kit (Promega) according to the manufacturer’s protocol. Luminescence was detected using a Veritas microplate luminometer (Turner Biosystems). All experiments were performed at least in triplicate, and results are presented as mean ± standard deviation.

### Quantitative RT-PCR.

Reverse transcription-PCR (RT-PCR) was used to quantitate cellular RNA and viral miRNAs in transfected and infected NHDF. Total RNA was isolated from infected cells using Trizol. cDNA was prepared using 100 ng of total RNA and either random hexamer primers or miRNA hairpin-specific primers (custom designed). Samples were incubated at 16°C for 30 min, 42°C for 30 min, and 85°C for 5 min. Real-time PCR (TaqMan) was used to analyze cDNA levels in transfected or infected samples. An ABI StepOnePlus real-time PCR machine was used with the following program for 40 cycles: 95°C for 15 s and 60°C for 1 min. miR-16, IKKα, IKKβ, CCL5, IL-6, and U6 primer/probe sets were obtained from ABI. HCMV miRNA primers and probes were custom designed.

### Enzyme-linked immunosorbent assay (ELISA).

ELISA Max kits for IL-6, CCL5, and TNF-α were obtained from BioLegend, and assays were performed according to the manufacturer’s instructions. NHDF, hAEC, and THP-1 cells (treated with tetradecanoyl phorbol acetate [TPA] for 24 h) were infected with WT or mutant viruses for 2 h (MOI of 3 for NHDF and 5 for hAEC and THP-1 cells). After this time, viral inoculum was removed and monolayers were replenished with fresh medium. At 48 and 72 h postinfection (NHDF and THP-1 cells) or 6 and 9 days postinfection (hAEC), supernatant was harvested, cell debris was removed, and the supernatants were frozen at −80°C until analysis. For transfected cell samples, NHDF were transfected with 30 pmol/well of miRNA/siRNA using 0.8 µl of RNAiMax, hAEC were transfected with 60 pmol/well miRNA/siRNA and 0.8 µl RNAiMax, and THP-1 cells were transfected with 40 pmol/well miRNA/siRNA and 2 µl RNAiMax in a 12-well dish. Seventy-two hours after transfection, medium was removed and replaced with fresh medium containing 2.5 ng IL-1β. Eight hours after IL-1β treatment, supernatants were harvested, and cell debris was removed and frozen prior to analysis. For IL-6 analysis, NHDF and hAEC supernatants were diluted 1:20. All other assays were performed using undiluted samples.

### Statistical analysis.

The Student *t* test (Microsoft Excel software) was used to determine *P* values. Results were considered significant at a probability (*P*) value of <0.05.
